# Epigenetic Modifications and Neuroplasticity in the Pathogenesis of Depression: A Focus on Early Life Stress

**DOI:** 10.3390/bs14100882

**Published:** 2024-10-01

**Authors:** Bianca Maria Benatti, Alice Adiletta, Paola Sgadò, Antonio Malgaroli, Mattia Ferro, Jacopo Lamanna

**Affiliations:** 1 Center for Behavioral Neuroscience and Communication (BNC), Vita-Salute San Raffaele University, 20132 Milan, Italy; b.benatti@studenti.unisr.it (B.M.B.); m.ferro@milano-sfu.it (M.F.); 2 Center for Mind/Brain Sciences, University of Trento, 38068 Rovereto, Italy; alice.adiletta@unitn.it (A.A.); paola.sgado@unitn.it (P.S.); 3 Faculty of Psychology, Vita-Salute San Raffaele University, 20132 Milan, Italy; 4 Clinical Center Tourette Syndrome, IRCCS Ospedale San Raffaele, 20127 Milan, Italy; 5 Department of Psychology, Sigmund Freud Private University, 20143 Milan, Italy

**Keywords:** major depressive disorder, early life stress, epigenetics, neuroplasticity, synaptic plasticity, childhood adversity, psychiatric disorders, methylation, histone, miRNA

## Abstract

Major depressive disorder (MDD) is a debilitating mental illness, and it is considered to be one of the leading causes of disability globally. The etiology of MDD is multifactorial, involving an interplay between biological, psychological, and social factors. Early life represents a critical period for development. Exposure to adverse childhood experiences is a major contributor to the global burden of disease and disability, doubling the risk of developing MDD later in life. Evidence suggests that stressful events experienced during that timeframe play a major role in the emergence of MDD, leading to epigenetic modifications, which might, in turn, influence brain structure, function, and behavior. Neuroplasticity seems to be a primary pathogenetic mechanism of MDD, and, similarly to epigenetic mechanisms, it is particularly sensitive to stress in the early postnatal period. In this review, we will collect and discuss recent studies supporting the role of epigenetics and neuroplasticity in the pathogenesis of MDD, with a focus on early life stress (ELS). We believe that understanding the epigenetic mechanisms by which ELS affects neuroplasticity offers potential pathways for identifying novel therapeutic targets for MDD, ultimately aiming to improve treatment outcomes for this debilitating disorder.

## 1. Introduction

Major depressive disorder (MDD) is a debilitating mental illness characterized by abnormalities of affect and mood, neurovegetative functions (e.g., appetite and sleep disturbances), cognition (e.g., inappropriate and persistent guilt and feelings of worthlessness), and psychomotor activity (e.g., agitation or retardation) [1]. MDD is one of the leading causes of disability worldwide [2], and it is linked to a higher likelihood of developing pathological conditions such as diabetes mellitus, heart disease, and stroke [3]. Moreover, it poses a risk of suicide. Suicidal ideation and suicidal behaviors can be symptoms of a depressive episode. In a recent meta-analysis, MDD was associated with significantly increased odds of suicide ideation, attempt, and death [4]. Although antidepressants represent the gold standard treatment for MDD, around 30% of the patients fail to achieve remission after two or more treatment trials with those drugs [5]. One reason for this could be that the patients fulfilling the Diagnostic and Statistical Manual of Mental Disorders 5th edition (DSM−5) diagnostic criteria may exhibit highly heterogeneous symptoms. This great dissimilarity between clinical manifestations may account not only for the difficulty in finding relevant causal effects of risk factors but also for the low beneficial effects of medical treatments [6]. Thus, the etiology of the disorder remains unclear. However, it is generally believed that MDD is a multifactorial syndrome caused by the complex interaction between biological, psychological, and social aspects [7].

A meta-analysis of twin studies sets MDD’s heritability to 37% [8], but the risk to develop the disorder is highly polygenic, involving many gene polymorphisms exerting small individual effects. More than 100 genetic risk loci have been linked to the disorder to date [9]. In this context, the complexity of identifying main genetic effects in genome-wide association studies (GWAS) could be due to the relevant genetic variants, leading to an increased risk only when exposed to adverse experiences. This phenomenon is called gene–environment (GxE) interaction [10]. One of the first crucial GxE interaction studies demonstrated that a higher risk of developing depression following stressful life events was found in individuals with two or more copies of the short allele of the serotonin transporter linked polymorphic region (5-HTTLPR) compared to those carrying the long allele [11], although a recent systematic review of the literature challenges this association [12]. A large amount of other G×E studies have shown analogous associations across various genes, environments, and phenotypes. For example, it was found that the relationship between maltreatment and antisocial behavior is modulated by the monoamine oxidase A (MAOA) genotype [13], while the catechol O-methyltransferase (COMT) genotype moderates the relationship between cannabis use and psychosis [14,15]. Therefore, researchers started questioning how different environmental factors could shape our behavior.

The discovery that gene activity can be regulated by our experiences has highlighted the intricate interplay between genetic and environmental factors, a concept leading to the definition of epigenetics [16]. Epigenetics is a promising, rapidly growing scientific field that studies the molecules and mechanisms that can perpetuate alternative gene activity states in the context of the same DNA sequence [17]. Over the past 50 years, there has been a growing interest in research and the applicability of epigenetics, expanding our knowledge in such fields [18,19].

Evidence suggests that stressful and/or traumatic events play a major role in the emergence of MDD psychopathology, especially if they occur during sensitive periods of life, such as early childhood [20]. During such periods, epigenetic programming is not only susceptible to environmental influences, like diet and toxins, but also maternal behavior or childhood abuse [21]. Early experiences can modify epigenetic markers and subsequent transcription patterns, influencing brain structure, function, and behavior. Following this discovery, it was found that early life stress (ELS) can disrupt the functional development of brain regions, neural circuits, and neurotransmitter systems crucial for cognition and affective behavior [22].

Theories on the modulation of plasticity processes during the development of the nervous system report that the effect of various environmental factors on the individual can be stronger if they occur during certain periods of lifetime, often referred to as “sensitive periods”, or “critical periods” [22,23]. Early postnatal life represents, along with embryonic development (prenatal environment), a major critical period for an individual [21]. Childhood, adolescence, and adulthood correlate to different peaks of inhibitory neuron development, synapse formation, synaptic pruning, and spine maintenance, whose dynamics can be dramatically altered by postnatal early life adversity (ELA) [22].

During the typical stress response, neuronal plasticity plays a crucial role in carrying adaptive intracellular changes within the brain [24]. Nonetheless, severe or chronic stressors can be harmful and could alter the ability of the brain to respond to stress [25]. Chronic exposure to stress can lead to long-term effects on brain structures [26,27]. Neuroplasticity has been suggested as a possible primary pathogenetic mechanism of MDD [28].

A great body of research on ELS or ELA and depression often relies on human studies. Clinical studies have proven useful to reveal the impact of adverse childhood experiences (ACEs) on psychopathology. The associations between ELS, health risk behaviors, and illness later in life have begun to be explored only quite recently. The first main study on this topic was conducted by Felitti and colleagues in 1998. Their research group showed, through the administration of the ACE questionnaire, that traumatic events experienced during childhood can increase the risk to develop alcoholism, drug abuse, depression, and many other non-psychologic or psychiatric conditions [29].

Exposure to ACEs (such as physical, psychological, and sexual abuse, or neglect household dysfunction [30]), is a major contributor to the global burden of disease and disability. A recent meta-analysis has gathered evidence from more than half a million adults in over 200 studies, and it attests that the prevalence of ACEs is 39.9% [31]. As of today, we know from both prospective and retrospective epidemiological studies that adverse experiences before age 5 years are most associated with the emergence of late childhood psychopathology symptoms [20]. The risk of developing MDD is indeed two times higher in subjects with a history of childhood trauma when compared to individuals who did not experience childhood adverse events [32]. Moreover, a meta-analysis by LeMoult and colleagues shows that some types of ELS are associated with a higher risk of developing MDD (such as physical, emotional, or sexual abuse), in contrast to other stressors (such as poverty, illness, and natural disasters) [33].

Many studies have gathered evidence on the epigenetic modifications in MDD patients who experienced ELS [34]. Human studies investigating epigenetic modifications in the context of ELA present some limits since they are vulnerable to temporality and recall biases and can only provide correlational inferences. To address this, preclinical studies play a crucial role in establishing cause–effect relationships, offering potential for therapeutic interventions. Understandably, no animal model developed until now perfectly reproduces the depression symptoms observed in humans. However, such models have enabled the discovery of several pathways that might contribute to the pathogenesis of the disease and have facilitated the study of the implicated molecular processes [22,35]. Just to make a few examples, the study of Darlene Francis demonstrated that modifications in maternal behavior early in the offspring’s life can lead to subsequent vulnerability to stress. Moreover, they found maternal behavior to be linked to hypothalamic–pituitary–adrenal (HPA) axis alterations, with maternally separated pups showing higher corticotropin-releasing factor (CRF) mRNA levels in the hippocampus and the amygdala, as well as higher CRF receptor binding in the locus coeruleus [36]. Other study worthy of mention shows that licking, grooming, and arched-back nursing promote hippocampal synaptogenesis and spatial learning and memory through increased expression of NMDA receptor subunit and brain-derived neurotrophic factor (BDNF) mRNA [37]. Thanks to a large number of animal and human studies, we now know that early life stress may result in enduring epigenetic marks in the genome [34]. Such marks alter gene expression and can result in the development of depressive symptoms by influencing neural and behavioral functions [38,39,40] (for an in-depth review on this topic, see [34]).

Thus, the aim of this review is to explore how ELS can alter neuroplasticity through epigenetic modifications and eventually lead to depression. To accomplish this, we will search for the most recent findings on the topic and structure them based on the main epigenetic mechanisms involved, exploring the two main forms of neuroplasticity (i.e., structural and functional synaptic plasticity). The former includes morphological changes (e.g., changes in the shape and number of dendrites and dendritic spine), as well as neurogenesis [41], while the latter consists of pre- and post-synaptic modifications, which may result in long-term potentiation (LTP) or long-term depression (LTD) of synaptic transmission [42,43].

## 2. Epigenetic Mechanisms in Major Depressive Disorder

Epigenetic processes are responsible for regulating gene expression and provide a bridge between the environment and the genome [44]. The three mainly studied epigenetic mechanisms comprise: (i) DNA methylation (DNAm); (ii) histone post-translational modifications (PTMs), with histone acetylation being the most important form; and (iii) regulation by noncoding RNAs, mainly micro-RNAs (miRNAs).

DNA methylation is widely recognized as a major epigenetic modification, and its main role is to inhibit gene transcription by transferring a methyl group (CH_3_) onto the C5 position of the cytosine to form 5-methylcytosine (5 mC) [45]. The reaction is catalyzed by DNA methyltransferases (DNMTs), a conserved family of DNA-modifying enzymes [46]. DNA methylation patterns are mostly erased and established during early mammalian development through a series of time frames of potential susceptibility to epigenetic dysregulation [21].

Regarding histone PTMS, it was only in 1974 that Kornberg and Thomas discovered that the histones organized DNA through the formation of repeating subunits (i.e., nucleosomes) [47]. Nucleosomes consist of 147 base pairs of DNA wrapped nearly twice around a protein core that contains the following two copies of four histone types: H2A, H2B, H3, and H4 [48]. Of the various histone PTMs, histone acetylation is one of the main studied mechanisms playing a role in depression. The acetylation of histones is performed by histone acetyltransferases (HATs), involving the transfer of an acetyl group (-COCH_3_) onto the N-terminal histone tails. Deacetylation, on the other hand, is catalyzed by histone deacetylases (HDACs). The activity and specificity of HATs can be modulated by a variety of mechanisms as follows: protein–protein interactions, protein cofactors, and autoacetylation [49]. There is abundant evidence that acetylation activates gene transcription, whereas methylation and ubiquitylation (the latter leading to even more profound changes in chromatin structure through the addition of large moieties) have variable effects depending on the precise residues and contexts. All histone PTMs are reversible. For example, HDACs remove added acetyl groups, and serine/threonine phosphatases remove phosphate groups [50].

Finally, miRNAs are considered one of the most significant discoveries of the last decade in molecular biology. They are small (∼20–30 nucleotide) noncoding RNAs that can regulate gene expression, acting as post-transcriptional repressors of gene expression in diverse biological contexts [51].

While these three epigenetic pathways have independent functions, they can also influence each other [52]. For instance, MeCP2, a methyl-CpG-binding protein, enlists the H3K9 (the 9th lysine residue of histone H3) methyltransferase SUV39H1 to target specific genes, resulting in H3K9 methylation and subsequent gene silencing [53]. Incorrect epigenetic marks can result in birth defects or even death. Epigenetics’ dysregulation is associated with different physiological conditions and diseases, including but not limited to aging, autoimmune diseases, cancers, neurodegenerative disorders, and psychopathology [52]. Epigenetic studies could offer insight into how environmental factors may influence the risk of depression by elucidating the role of heritable DNA methylation patterns [54]. These patterns may contribute to the heritability of depression [55]. At the same time, epigenetic mechanisms are also known to be modulated by environmental exposure [56].

Monozygotic twin studies found that twins develop epigenetic differences over the years, possibly due to the distinct life experiences throughout development [57]. Interestingly, several studies have shown consistent epigenetic changes in the brains of animal models of depression as well as in post-mortem brain samples of MDD-affected patients [38,58,59,60]. Animal models of depression show that the development of depressive-like behaviors is linked to enduring changes in gene regulation, which are similar across different stress paradigms [35]. To note, some studies found variations in the DNA methylation of the CRF gene and BDNF in animals that experienced stressful experiences, as well as differences in histone acetylation modulating cFOS (an immediately early gene activated by action potential firing in neurons [61]) and BDNF gene expression, and alterations in HDAC2 [62,63,64,65]. Epigenetic changes related to stress have also been shown to affect neurotransmitter systems that are critically involved in the development of depression and anxiety, including serotoninergic [66,67], dopaminergic [68], glutamatergic [69,70], and GABAergic [70,71].

For what concerns human studies, the most consistently replicated results in MDD involve DNA methylation. A recent meta-analysis found significant differences in DNA methylation in the prefrontal cortex (PFC) and cerebellum of the brains of patients with MDD who committed suicide, compared to non-psychiatric controls [59]. Another meta-analysis worthy of note reported differentially methylated positions (DMPs) and regions (DMRs) associated with the disorder. Importantly, their findings indicate that the pathways involved are linked to mechanisms of neuronal synaptic plasticity and inflammation [58].

## 3. The Role of Neural Plasticity in Depression

Neuroplasticity consists of functional and structural changes in the brain that allow for adaptation to the environment [52], as well as for learning, memory, and recovery following a brain injury [72]. Alterations in brain plasticity are supported by evidence of changes in neurotrophic factors; specifically, BDNF appears to play a major role in MDD [73]. Human post-mortem studies have revealed a decrease in BDNF [74] in the hippocampus of patients suffering from depression. BDNF primarily binds to tropomyosin receptor kinase B (TrkB) receptors, facilitating neuronal differentiation and growth, and the decrease observed in depression may contribute to the reduction in hippocampal volume seen in depressed patients [38]. Furthermore, the BDNF/TrkB signaling was found to be upregulated by both classic and rapid-acting antidepressants [75,76,77].

The neuroplasticity hypothesis in MDD has been investigated by clinical studies thanks to the use of rapid-acting antidepressants such as ketamine and other NMDA receptor antagonists. These drugs were found to increase synaptic number and activity in the PFC [78,79,80] and to produce rapid antidepressant effects [42]. Research using rodent models has further validated findings in humans, demonstrating that stress exposure leads to neuronal and glial atrophy and loss in both the PFC and hippocampus, similar to the effects observed in MDD [81]. Moreover, it was demonstrated that part of the antidepressant effect of ketamine happens via the dopamine (DA) system, as it restores the reduced activity of DA neurons and enhances long-term potentiation in the hippocampus-accumbens pathway, partially through the activation of D1 receptors [82]. Finally, recently validated treatments for drug-resistant forms of MDD are based on transcranial magnetic stimulation protocols, which are believed to ameliorate symptomatology through the recruitment of synaptic plasticity processes [83,84].

In a recent study by our group, it was found that DA-dependent LTP (DA-LTP) is occluded in rats vulnerable to chronic mild stress (CMS), possibly reflecting differential expression of AMPA receptors [85]. This occlusion in plasticity is not observed in rats treated with ketamine, which seems to rescue both medial PFC (mPFC) plasticity and cortico-limbic synaptic drive, likely mediated by the promotion of ventral tegmental area (VTA) activity [85]. Conversely, we previously showed that DA-LTP in mPFC is enhanced by acute forms of mild stress [86], suggesting a U-shaped relationship between stress dynamics and such forms of neural plasticity. Neuromodulators, including metabotropic monoamine systems (such as dopamine, serotonin, and noradrenaline), which represent the typical targets of antidepressants, greatly modulate plasticity thresholds and characteristics [87].

D1-type and D2-type receptors exert opposite action on neuronal activity as well as synaptic plasticity [88]. D1-like receptors appear to cause disinhibition and modulation of NMDAR signaling [89]. On the contrary, D2 receptors seem to reduce protein kinase A (PKA) activity and limit Ca^2+^-dependent signaling mechanisms [90,91]. Importantly, the D2 family of DA receptors is present in the post-synaptic membranes of PFC pyramidal neurons [92]. In 2017, Robinson and Sohal proposed a model in which the D2 receptors act via Gs-mediated signaling and not Gi/o-mediated signaling, stimulating PKA and leading to excitability in the PFC [93]. Recent studies better investigated this aspect, showing that both canonical and non-canonical signaling pathways are triggered by D2 receptor activation in PFC and play a role in the effects exerted by early-life stress [94] and social behavior [95]. Furthermore, D2 receptor activation results in endocytosis, which was found to increase spine formation and neuronal activity in the striatum [96].

The sustained modifications in synaptic structure and function involved in neuroplasticity require de novo gene expression. This de novo transcription is orchestrated by chromatin and epigenetic modifications [97]. Moreover, epigenetic modifications can influence neuroplasticity by altering the levels of neurotrophic factors such as BDNF [98,99,100]. It has been demonstrated that ELA can lead to abnormal BDNF methylation and mRNA expression patterns in the hippocampus, PFC, and amygdala [75,101].

Some preclinical studies, together with human studies, suggest that dysregulation of neuronal and synaptic plasticity caused by chronic stressful life events may contribute to the pathophysiology of MDD [60]. If we focus on the early postnatal period, research shows that neural circuits are being formed and refined during such a time frame [23]. Neuroplasticity is extremely active during pre- and postnatal brain development, and circuit formation undergoes an interplay of synaptic potentiation/depression, synaptogenesis, synaptic pruning, myelination, and neurogenesis. Thus, early life stress (ELS) can modify the functional development of brain regions, neural circuits, and neurotransmitter systems crucial for cognition and affective behavior [22].

## 4. Histone Modifications, Neuroplasticity, and Depression

Histone acetylation was first linked to depression through systemic or intracerebral administration of HDAC inhibitors (HDACis) in animal models [38]. HDACi, both alone and in combination with antidepressants, showed enhanced antidepressant responses [102,103,104]. For example, the infusion of a specific class of HDACi (MS275) into the nucleus accumbens (NAc), a key region involved in MDD and an important area of the reward system [105], exerts an antidepressant effect [104].

A recent study by Sun and colleagues [106] reproducing an ELS rat model of post-traumatic stress disorder (PTSD) using a maternal separation (MS) protocol and a subsequent single prolonged stress (SPS) procedure examined H3K9 acetylation (H3K9ac), HDAC2, and BDNF levels in the hippocampus. Even though the study doesn’t specifically use an animal model of depression, MS and SPS can elicit a variety of symptoms, including anxiety and depression. The authors evaluated depression through the forced swim test (FST) and assessed anxiety with the open field test (OFT) and the elevated plus maze test (EPMT). The results show that rats who experienced 6 h of MS (MS6h-PTSD) exhibited increased anxiety, depression, and contextual fear memory compared to those who had experienced only 3 h of MS (MS3h-PTSD). Moreover, female MS6h-PTSD rats showed lower BDNF mRNA and protein expression. However, in male rats, a relatively mild MS3h-PTSD had a protective effect against SPS experienced in adulthood, increasing H3K9ac and BDNF gene expression. On the other hand, MS6h-PTSD led to reduced BDNF mRNA levels and worse outcomes. Interestingly, this group had the lowest H3K9ac levels among all male rats. Sun and his team interpreted their findings, suggesting that the MS3h procedure promoted the recovery of H3K9ac levels in male rats after SPS. On the other hand, the longer duration of MS experienced by MS6h-PTSD rats might have led to further loss of acetylation and worse outcomes relative to rats that only experienced the SPS procedure in adulthood [106]. As for HDAC2, increasing the duration of MS was linked to higher mRNA expression of HDAC2 in both sexes. In line with these results, Sun and colleagues found measures of synaptic plasticity to be more disrupted in the MS6h-PTSD groups, which was especially apparent in indicators such as the width of the synaptic cleft and the thickness of post-synaptic density [106]. HDAC2 has been identified as a regulator of synaptic and neuronal plasticity. A heightened HDAC2 expression decreases dendritic spine density, synapse number, synaptic plasticity, and memory formation [107]. Moreover, the administration of class I HDACi CI-994 downregulates HDAC2/3 and has antidepressant effects [108]. Previous studies on CI-994 also found that it reverses MS-induced GABAergic alterations, increasing H3K9ac and normalizing expression levels of the postsynaptic scaffolding A-kinase anchoring protein 150 (AKAP150) in the VTA. AKAP150 controls GABA_A_ receptor trafficking in VTA dopamine neurons [34,109,110].

Gaszner et al. [111] reproduced a 3-hit model of depression on pituitary adenylate cyclase-activating polypeptide (PACAP) heterozygous male mice. They found that mice that were exposed to 3h of maternal deprivation (MD) and that received vehicle injection showed low H3K9ac immunosignal in the 3 hippocampal areas of study (CA1, CA3, and dentate gyrus (DG)), but a subsequent adult exposure to chronic variable mild stress (CVMS) increased H3K9ac. This result is in line with the previously cited study by Sun et al. [106], who interpreted the higher levels of H3K9ac in male rats that experienced adulthood stress after 3 h of MS in early life as a possible protective factor. Furthermore, Gaszner et al. [111] studied the effect of fluoxetine, which had an acetylation-increasing effect in all groups, except for the subgroup that was exposed to MD and CVMS. The selective serotonin reuptake inhibitors (SSRIs) did not affect the H3K9ac in CA1, and it surprisingly reduced histone acetylation in CA3 and DG [111].

Another study from Jiang and colleagues in 2021 [99] investigated BDNF expression and H3K9me2 (dimethylation of histone 3 at lysine 9) in the hippocampus and mPFC and their relationship with depressive-like behaviors. H3K9me2, a key epigenetic mark of gene expression silencing, has been proposed as a possible mechanism underlying decreased BDNF expression and the impairment of emotion and cognition. The results of the study show that chronic unpredictable mild stress (CUMS) induces depressive-like behaviors in rats and results in increased H3K9me2 expression and concomitant decreased BDNF expression in the hippocampus and mPFC. Most importantly, MS rats (3 hours) experiencing CUMS (MS + CUMS) have more severe depressive phenotypes, higher expression of H3K9me2 in the hippocampus and mPFC, and lower expression of BDNF in the hippocampus and mPFC. In addition, administration of the G9a inhibitor, known to reduce H3K9me2, reverses most of these alterations [99]. The study by Jiang and colleagues did not find the same protective effect of MS + adulthood stress found in the studies mentioned above. Notably, the authors conducted their experiments on different animals (PACAP mice, Sprague Dawley rats, and Wistar rats) with different ELS paradigms. For example, while they all used a 3-hour MS/MD paradigm, Jiang et al. maternally separated rats from postnatal day (PND) 8 to 21, while Sun et al. did it on different postnatal days (PND2-PND14). Moreover, they all used different adult stress paradigms.

MS in early life has negative effects on epigenetic mechanisms associated with decreased p11 (S100A10) expression. P11 plays an important role in depression, as it interacts with the 5-hydroxytryptamine (5-HT) 1B receptor by increasing its trafficking to the cell surface, where it binds to serotonin [112]. In their study, Seo, Lee, and colleagues (2021) found that histone acetylation at the hippocampal p11 promoter was reduced in both young adult and middle-aged mice [113]. Reduced hippocampal p11 expression was associated with significant decreases in H3 acetylation and tri-methylation of histone 3 at lysine 4 (H3K4me3), a marker of histone modification activation at the p11 promoter region, and with a significant increase in tri-methylation of histone 3 at lysine 27 (H3K27me3), a marker of histone modification repression. Moreover, p11 expression, H3 acetylation, and H3K4me3 after MS were more severely altered in middle adulthood compared to young adult mice [113]. In another study, Seo, Choi, and colleagues [114] found that an early enriched environment (EE) prevented depressive-like behaviors induced by chronic unpredictable stress (CUS) during adulthood, increasing H3 acetylation, restoring H3K4me3 levels, and decreasing the H3K27me3 levels within the p11 promoter. Furthermore, while CUS increased HDAC5 expression, the EE prevented this increment.

Another recent study investigated whether EE could prevent negative behavioral and epigenetic effects in rats who experienced MD. The authors suggest that EE, especially in the long term (40 days), can prevent epigenetic changes caused by ELS in female and male rats. Offspring deprived of maternal care showed increased HDAC and DNMT activities (see in the next paragraph). However, such effects were dependent on sex and developmental period, but prolonged exposure to EE could reverse this increase. Noteworthy, females were more susceptible to epigenetic changes induced by MD in PFC, and an EE of 20 days showed more epigenetic modifications in the hippocampus of female rats rather than male rats [115]. Finally, Baghel and colleagues found that a poly (I:C) (a semisynthetic double-stranded RNA and a potent stimulator of the immune system) injection at PND7 subsequently increased H3K9 acetylation in the promoter of BDNF, Arc, and Egr1 genes and later (at 12 weeks) decreased it in the frontal cortex and hippocampus of exposed rats [116]. Notably, long-term effects of poly I:C were previously found to impair spatial and fear conditioning memory through changes in the synaptic plasticity gene expression (BDNF, Arc, and Egr1) in the same brain regions. The authors also found enhanced tumor necrosis factor alpha (TNF-α) positive microglia cells in the hippocampus, and interestingly, TNF-α-positive cells were less in number in 12-week rats as compared to early age (3-week) [117]. TNF-α level might be playing a crucial role in the reduction or increase in DNA methylation at different time points. Initially, in 3- and 6-week rats, when the TNF-α level is high, DNA methylation is reduced, whereas it appears to increase in 12-week rats, when the TNF-α level is relatively low [116].

## 5. DNA Methylation, Neuroplasticity, and Depression

Interesting results also come from DNA methylation. ELS results in global alterations in DNA methylation and DNMT levels that can be observed throughout the lifespan and even into following generations [34]. Interestingly, from a study investigating contextual fear conditioning (CFC) in mice, we now know that CFC leads to extensive DNA damage in a cluster of hippocampal CA1 neurons, activating the toll-like receptor 9 (Tlr9) signaling pathway and accumulating centrosomal DNA damage repair complexes (DDRs) [118]. While Tlr9 signaling was found to be generally induced by unmethylated CpG DNA sequences of bacterial origin, demethylation of mammalian DNA during LTP and learning can also lead to Tlr9 pathway activation [119,120]. It was also found that Tlr9 plays a critical role in the maintenance of neuronal genomic integrity, as the knockdown of Tlr9 impaired memory and reduced CFC, leading to genomic instability and cognitive impairments typically observed in accelerated senescence and psychiatric and neurodegenerative disorders [118].

Methylation of the *Oxtr* (oxytocin receptor) gene might play an important role in ELS-induced susceptibility to depression in adult mice. Oxytocin has anxiolytic effects, and the *Oxtr* gene was also found to be regulated via histone acetylation. LPM570065, a novel 5-HT, norepinephrine (NE), and DA-triple reuptake inhibitor, can reverse depressive-like behaviors, methylation of the *Oxtr* gene in the hippocampus, and the increased protein expression of Dnmt1 and Dnmt3a in mice that experienced MS and social defeat stress (SDS). Moreover, LPM570065 was found to increase the density of dendritic spines in hippocampal CA1 neurons [121].

An interesting study by Catale et al. [122] found the following two distinct types of ELS experiences: early social isolation (ESI) and early social stress (ESS), associated with depression-like and addiction-like behavior in adult mice, respectively, induce different and permanent alterations in DNA methylation in brain and blood samples. From their findings, mice exposed to ESI showed more drastic and widespread changes compared with ESS and control mice, with a general and substantial increase in global DNA methylation levels in the striatum (in both the dorsolateral and dorsomedial subregions), NAc (core and shell), amygdala (basolateral and central), and hippocampus (CA1, CA3, and DG). Moreover, exposure to ESI determined expression changes in five of seven genes (Dnmt1, Dnmt3a, Dnmt3b, Mecp2, and Tet1), whereas ESS mice showed expression changes in only three genes. The authors also unexpectedly measured a drastic decrease in DNA methylation levels in microglia in the ESI group. Microglia plays an important role in synaptic maturation and in shaping brain circuits during development, and it has been found to be particularly sensitive to stress during critical developmental periods [123]. Catale and her team hypothesize that these alterations in microglia could represent a mechanism through which microglia holds memories of the ESI, becoming more reactive. In support of this hypothesis, the pharmacological inhibition of ELS-induced microglial activation has been shown to rescue the ELS-induced depressive-like phenotype in adulthood [124]. Wang et al. (2020) found that this could happen through the inhibition of high mobility group box-1 (Hmgb1), an alarmin protein that can prime proinflammatory immune responses [125]. Most importantly, Catale et al. (2020) found that peripheral epigenetic alteration was consistent with the changes in DNA methylation of microglia rather than with the changes in other brain cell populations [122]. Clinical data of DNA methylation measured in MDD patients demonstrate an effect of the diagnosis but not of the childhood abuse/neglect experience. However, the DNA methylation levels were assessed in patients during antidepressant treatment, which might have affected the results [122].

Another study from Guo and colleagues (2022) investigated the effect of DNA methylation of the dopamine D2 receptor (Drd2) in the VTA on ELS-induced depression in adult rats [126]. From their research, higher levels of depressive-like behaviors correlated with lower levels of Drd2 protein and mRNA expression in the VTA of the MD and MD + CUS groups, compared to controls. On the other hand, rats who only experienced adult CUS showed increased Drd2 expression. Finally, levels of *Drd2* promoter methylation were significantly higher among the three groups (CUS, MD, and MD/CUS) compared to controls. The study shows some following limitations: for example, the role of DNMTs has not been investigated, and the authors did not assess Drd2 expression on different neuronal subpopulations. Drd2 expression on non-dopaminergic neurons can exert various effects on plasticity. In fact, postsynaptic D2 receptors on the VTA GABAergic neurons can exert an inhibitory input onto dopaminergic neurons [126].

The influence of D2-type receptors on plasticity is still under investigation. Oh et al. (2021) found increased excitatory synapse density on prefrontal pyramidal neurons following ELS, and they were able to determine that this increase was not dependent on D1 receptors but was instead affected by D2 receptor signaling. They concluded that D2 receptors play a selective role in mediating synaptic changes in the PFC following ELS [127]. Thus, it would be interesting to clarify how the methylation of the *Drd2* promoter cited in the previous study [126] could regulate the correlated activities of D2-type receptors in the context of neuroplasticity.

Investigating the role of DNMTs could be interesting in understanding how DNA methylation can be influenced by stress. One study by Urb and colleagues (2019) examined the link between glucocorticoid receptor (GR) signaling and the expression of DNMTs in an ELS paradigm. They found that, after MS, Dnmt1, Dnmt3a, and Dnmt3b expression and DNMTs activity were markedly upregulated in the PFC of Wistar male rats. Furthermore, MS was shown to upregulate Dnmt3b expression via GR binding at the *Dnmt3b* promoter in the PFC. Nonetheless, other unknown transcription factors could participate in the regulation of Dnmt3a expression. Thus, the authors conclude that the intermittent increases in corticosterone after MS are an essential factor in heightened DNMT levels, and the increase in DNMT mRNA may be mediated by GR [128].

As we previously said, most of the time the different epigenetic mechanisms act concomitantly. Some of the studies cited in the histone PTMs paragraph, for example, investigated the role of DNA methylation together with histone modifications. The following are a few examples: the study from Seo, Lee, and colleagues (2021) showed that p11 expression, H3 acetylation, and H3K4me3 after MS were more severely altered in middle adulthood compared to young adult mice. Together with these results, they also found that increased DNA methylation in p11 promoter regions was only present in middle-aged and not in young adulthood mice. The increased DNA methylation, together with the altered histone modifications, could account for the reduced p11 expression in middle-aged MS animals [113]. Another example is the study by Borba and colleagues (2021), which found heightened HDACs and DNMTs in MD female rats’ PFC and hippocampus, as well as in the hippocampus of male rats [115]. Interestingly, in male rats, only exposure to an EE for 40 days could decrease these levels, while females needed less EE time (20 days) to produce a reduction in the hippocampus [115].

Interesting human data on childhood trauma and DNA methylation come from the Avon Longitudinal Study of Parents and Children (ALSPAC) prospective cohort [129]. The authors report associations between childhood adversity and DNA methylation across the lifetime, suggesting that early childhood (between 3 and 5 years old) might be a sensitive period for the biological embedding of childhood adversity that manifests in adolescence (15 years old). Many of the genes with differential methylation patterns that were identified have previously been associated with processes that may influence subsequent disease outcomes. For example, *CUX2* is responsible for encoding a transcription factor that plays a role in dendrite and synapse formation [130]. *CUX2* modifications could potentially affect neurodevelopment and increase susceptibility to mental disorders. Lussier and colleagues hypothesize that “sleeper” subtle desynchronization patterns of DNA methylation levels, which evade early detection but compare later in life, may explain why diseases emerge years after exposure to risk factors. Furthermore, most of the top loci found showed little individual-level stability over time. Thus, it would be interesting to further investigate these epigenetic modifications through adulthood as well [129].

## 6. Noncoding RNAs, Neuroplasticity, and Depression

Noncoding RNAs (ncRNAs) have been recently identified as a possible breakthrough link between depression and neuroinflammation [131]. MiRNAs have emerged as possible regulators of neural plasticity and higher brain functioning. Mckibben and colleagues in 2021 presented the first study to investigate the effect of genome-wide MS on miRNAs in three brain regions (PFC, amygdala, and hippocampus), accounting for sex effects in rats [132]. While other studies found that ELS produced differences in miRNA expression [133], almost no previous study assessed both male and female subjects. The authors demonstrated that MS produced depressive-like behaviors, altering seven PFC, nine amygdala, and seven hippocampal miRNAs, and sex moderated the effect of MS on several miRNAs’ expression.

MiRNA-200b-5p, miRNA-196b-5p in the hippocampus, and miRNA-34c-3p in the amygdala, all significantly upregulated, were the most affected by MS. Moreover, gene ontology analyses revealed that sex altered the effect of MS on miRNAs involved in synapse organization, behavior, and negative regulation of nervous system development, and the greatest impact of sex on MS-induced miRNAs was found in the PFC and hippocampus. Furthermore, the authors compared their MS-altered miRNAs with some previously reported miRNA alteration in animal models of depression and in MDD patients, reporting mixed results. This could be due to the different brain regions studied and to the central versus peripheral miRNA analyses, which often do not overlap. Finally, Mckibben and Diwedi found that MS males exhibited a network of mostly positive (82%) correlations between miRNAs across brain regions. In females, MS decreased the total number of significant correlations between miRNAs in the hippocampus and the amygdala and increased the number of negative correlations between miRNAs in the PFC and in the hippocampus (41%). As the authors point out, these results should be compared with other ELS and adult stress paradigms to verify if these miRNA changes reflect a specific effect of MS [132]. In a subsequent study, the two authors investigated sex differences in hypothalamic miRNAs and gene expression changes following MS and adult stress (restraint stress, RS) [134]. The results show that both MS and RS produce significant miRNA alterations, but RS does not seem to precipitate in depressive-like behaviors in MS rats. Sex differences were also demonstrated, with males expressing greater miRNA changes after MS. Only the behavioral performance at the FST and 6 miRNAs (some of which localized on the X chromosome) were affected by estrus timing. Thus, estrus phase does not consistently contribute to stress susceptibility. Interestingly, MS also led to an increased expression of Mapk6 (mitogen-activated protein kinase 6) and Mmp19 (matrix metalloproteinase-19). MiRNA-gene target mapping revealed several miRNA regulatory hubs, including miRNA-301b-3p, -132-3p, -132-5p, -449a-5p, -30e-5p, -338-3p, -144-3p, and let7 g-3p. Gene ontology analysis confirmed that these miRNA gene targets were significantly involved in MAPK signaling. Finally, the authors applied an EE, which had a strong effect on animal behavior and miRNA expression and even reversed some of the effects of MS, especially in the MAPK signaling pathway. Some of the altered miRNAs shown in this study have been previously studied by other authors, reporting similar results. MiRNA-132 and miRNA-124 are mostly restricted to the nervous system and are key to brain development through their roles in neuronal differentiation (miRNA-124) and morphogenesis (miRNA-132) [133]. Even though there are some contrasting results regarding its activity, most of the evidence seems to point to a negative effect of increased miRNA-124 in depression [135,136,137,138]. More specifically, adult and ELS stress paradigms generally increase hippocampal and PFC miRNA-124 levels, which have been frequently linked to reduced levels of BDNF [136,137,138].

However, in contrast, Higuchi et al. (2016) found that CUMS led to reduced miRNA-124 and that a viral-mediated miRNA-124 overexpression in hippocampal neurons conferred behavioral resilience to the stressed mice group, whereas an inhibition led to greater behavioral susceptibility to a milder stress paradigm [139]. Moreover, Karen and colleagues found that ELS downregulates miRNA-124a and upregulates miRNA-132 expression, and these changes were brought back to normal with a Lactobacillus supplement [140].

One possible explanation of this apparently contradictory data could be that miRNAs dynamically change their expression levels in response to stressors. Observing hippocampal miRNA-124 at different stages of CUMS-induced depression [141] revealed that the differences at various stages of CUMS may be caused by changes in stress resilience or stress susceptibility. From 5 to 6 weeks after CUMS, miRNA-124 levels are high, and depression-like behaviors are present but not stable, possibly indicating stress resilience in rats. However, from 7 to 8 weeks, with the continuous presence of stress, miRNA-124 starts to decline, neurogenesis dysfunctions appear, the number of neurons in the DG decreases, and depression-like behaviors become more stable.

In addition, a study identified a strong interplay between miRNA-342 and TNF-α in potentiating microglial activation, associating its expression with depressive-like behaviors for the first time [142]. As we previously mentioned, microglial activation is currently being studied as a possible mediator of depressive-like behaviors following ELS. In addition to the cited decrease in DNA methylation in microglia after ESI found by Catale and colleagues [122], Bràs et al. (2022) found that high-corticosterone stress-responsive (H-CSR) rats exposed to peri-pubertal stress (PPS) showed an activated microglial status (with decreases in microglial arborization volumes and number of branches) in the hippocampus. This particular group of ELS-exposed rats (H-CSR) also exhibited increased depressive-like behaviors and increased levels of hippocampal Iba-1, TNF-α, and miRNA-342. Interestingly, hippocampal TNF-α expression was positively correlated with the worsening of depressive-like behaviors (as shown by FST) [142].

ELS also seems to alter miRNA (miRNA-135a and miRNA-16) expression in the mPFC in a sex-dependent manner. In particular, ELS reduced miRNA-135a in females and miRNA-16 in males, and subsequent fatty acid amide hydrolase (FAAH) inhibition by URB597 during post-adolescence restored these effects and the depressive-like phenotype. However, if one considers the different brain areas examined, the authors also found increased miRNA-135 in the lateral habenula (LHb) and dorsal raphe of female ELS rats, and paroxetine downregulated their expression. ELS also downregulated miR-16 in the LHb, and treatment with URB597 and paroxetine elevated its expression. No correlation was observed between LHb-miR-16 expression and behavior [143].

The human literature on how ELS relates to miRNA is still quite limited, mostly overlapping with various psychiatric disorders. Since miRNAs themselves can be regulated through epigenetic modifications, two studies have explored methylation patterns on miRNA promoter regions in relation to ELS. In an all-male sample from low socioeconomic status and high child abuse backgrounds, patterns of altered methylation in promoter regions of 31 miRNAs were found [133,144].

## 7. Conclusions

**Figure 1:**
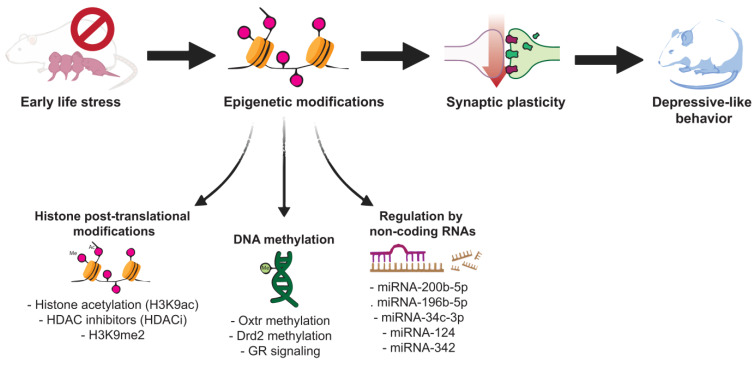
Scheme illustrating the main epigenetic mechanisms that might play a role in the development of major depressive disorder following early-life stress.

The purpose of this review was to gather some of the most recent studies investigating how early-life, postnatal stressful experiences can result in epigenetic modifications and disruptions in neuroplasticity, leading to depressive-like behaviors in various animal models and the link with MDD. As pointed out throughout the paper, there are numerous mechanisms involved in MDD, and investigating its underlying causes can be challenging. Evidence that epigenetic changes contribute to MDD pathophysiology has accumulated rapidly over the last decade (Figure 1 provides a summarizing scheme of the involved mechanisms).

Thus, identifying epigenetic mechanisms could expand our knowledge of the disorder. According to recent animal models of ELS, depressive-like behaviors and changes in epigenetic mechanisms are often observed when exposing mice or rat pups to MS and/or MD or a series of chronic stress paradigms. Moreover, from what we’ve gathered, ELS can lead to disruption in neuroplasticity measures through a variety of epigenetic modifications, especially those targeting BDNF.

Two unsolved questions concerning MDD involve its chronic nature and the delayed response to antidepressant treatments commonly observed in patients. The prolonged course of MDD might stem from gradual yet enduring adaptation processes facilitated by various epigenetic mechanisms [60].

Some of the limits that commonly characterize the existing research on the pathophysiology of MDD are as follows: (i) a scarce number of studies investigating sex-based differences in depression, especially in animal models; (ii) a limited replication of the results and overlapping of the brain regions under investigation; and (iii) animal models of depression still need to be unambiguously defined.

While sex differences may be present in the etiology and pathogenesis of MDD [145,146], many studies are still being conducted uniquely on male rodents. Understanding the molecular mechanisms involved in such differences is of great importance; thus, more research should be conducted on both male and female rats. Some of the articles included in this review highlighted sex differences in various epigenetic modifications following ELS. For example, it appears that a shorter MS/MD duration may produce a protective effect on male pups but not on females [106,111]. However, as previously pointed out, such evidence was not replicated by another study from Jiang and colleagues [99]. Moreover, sex was found to moderate the effect of MS on several miRNAs’ expression and was reported to produce greater miRNA changes in males [132,133,134,142]. Another study also reported female rats to be more susceptible to epigenetic changes induced by MD in the PFC, and when exposed to an EE, they also showed more epigenetic modifications in the hippocampus compared to male rats [115].

The second point (ii) deals with the topic of replicating existing experiments. The scientific literature on this topic is still in its early stages; hence, a growing number of studies replicating previous experiments and examining the same brain regions are needed. The third and final point (iii) states that animal models of depression still need to be unambiguously defined. A series of rodent studies of depression have been cited in this review, and most of them adopted a peculiar stress paradigm that was quite different from the other ones in terms of stressor type, duration, and developmental timing of the animal studied. This discrepancy surely demonstrates that different chronic stress paradigms at different developmental stages could lead to depressive-like phenotypes, but it does not help the process of replication (point ii).

In conclusion, the evidence gathered in this review seems to leave a few doubts about the importance of epigenetics and early-life stress in the pathogenesis of psychiatric disorders such as MDD. In fact, these epigenetic changes seem to impact a wide range of mechanisms that have been found altered in MDD, such as neuroplasticity, inflammation, neuromodulatory pathways, and others. All this evidence led to the research of new, rapid-acting “epidrugs”, such as the HDAC inhibitors mentioned above. Clearly, clinical trials on humans with MDD are still out of sight, but with the development of novel and highly selective compounds, HDACis remain one of the most promising avenues for the future treatment of this dramatic disorder.

## Data Availability

Data is contained within the article.
